# Time to onset in statistical signal detection revisited: A follow‐up study in long‐term onset adverse drug reactions

**DOI:** 10.1002/pds.4790

**Published:** 2019-06-12

**Authors:** Joep H.G. Scholl, Florence P.A.M. van Hunsel, Eelko Hak, Eugène P. van Puijenbroek

**Affiliations:** ^1^ Netherlands Pharmacovigilance Centre Lareb 's‐Hertogenbosch The Netherlands; ^2^ Department of PharmacoTherapy – Epidemiology & ‐Economics University of Groningen Groningen The Netherlands

**Keywords:** ADR, latency, pharmacoepidemiology, pharmacovigilance, signal detection, time to onset

## Abstract

**Purpose:**

In a previous study, we developed a signal detection method using the time to onset (TTO) of adverse drug reactions (ADRs). The aim of the current study was to investigate this method in a subset of ADRs with a longer TTO and to compare its performance with disproportionality analysis.

**Methods:**

Using The Netherlands's spontaneous reporting database, TTO distributions for drug—ADR associations with a median TTO of 7 days or more were compared with other drugs with the same ADR using the two‐sample Anderson–Darling (AD) test. Presence in the Summary of Product Characteristics (SPC) was used as the gold standard for identification of a true ADR. Twelve combinations with different values for the number of reports and median TTO were tested. Performance in terms of sensitivity and positive predictive value (PPV) was compared with disproportionality analysis. A sensitivity analysis was performed to compare the results with those from the previous study.

**Results:**

A total of 38 017 case reports, containing 32 478 unique drug—ADR associations. Sensitivity was lower for the TTO method (range 0.08‐0.34) compared with disproportionality analysis (range 0.60‐0.87), whereas PPV was similar for both methods (range 0.93‐1.0). The results from the sensitivity analysis were similar to the original analysis.

**Conclusions:**

Because of its low sensitivity, the developed TTO method cannot replace disproportionality analysis as a signal detection tool. It may be useful in combination with other methods.

**KEY POINTS**

- Disproportionality analysis, although the most commonly used method in statistical signal detection of adverse drug reactions (ADRs), has its limitations.
- A previously developed method using time to onset (TTO) in signal detection had a low sensitivity, mainly due to an overrepresentation of ADRs with a short TTO. The current study with a subset of ADR with a longer TTO showed similar sensitivity to our previous study with good positive predictive value (PPV).
- TTO‐based signal detection cannot replace disproportionality analysis and should be further investigated in combination with other signal detection methods.

## 1 INTRODUCTION

Statistical signal detection on databases containing spontaneous reports of adverse drug reactions (ADRs) has become a valuable addition to case‐by‐case assessment of individual case reports. Historically, these statistical methods are mainly based on observed versus expected ratios, using both Bayesian and frequentist approaches.^1^ In recent years, additional methods have been investigated and developed as a way to generate potential signals, including the use of the time to onset (TTO).^2^, ^3^, ^4^, ^5^, ^6^, ^7^, ^8^, ^9^, ^10^ In general, the TTO studies are based on the hypothesis that the TTO distributions differ between true causally related ADRs and drug—event combinations without a causal relationship. This hypothesis makes sense from a pharmacological point of view since ADRs can have a different time course (rapid, first dose, early, intermediate, late, and delayed).^11^ However, contradictory results regarding the additional value of TTO analyses in signal detection have been reported in different studies.^3^, ^5^, ^10^

Recently, we investigated the performance of a TTO‐based method and compared it with disproportionality analysis based on the reporting odds ratio (ROR) used at our centre.^10^ The main finding was that the sensitivity was too low to be useful for screening, most likely because of two main reasons: (a) most ADRs in our database have a median TTO of approximately 1 to 2 days, resulting in decreased discriminative power. This over representation may have a pharmacological cause since the majority of ADRs are type A effects, and their TTO is related to the pharmacokinetic/pharmacodynamics properties of the suspect drug.^12^ On the other hand, it may also be the result of selective reporting. Indeed, recall bias is likely to occur with an increasing TTO since the reporter does not associate the complaints with previous drug exposure. Additionally, coincidental events with a short latency may be reported unjustly and may therefore be misclassified as true ADRs. (b) TTO distributions of drug—ADR combinations were tested against all other ADRs for the same drug (drug—ADR_other_) and against all other drugs for the same ADR (drug_other_—ADR). For a drug—ADR combination to be a true positive signal, both test results had to be statistically significant. This may however, have been an overly conservative approach, and one could debate whether the comparison with drug_other_—ADR only would be more appropriate. After all, the hypothesis is that the TTO of a true ADR for a certain drug will have a different distribution compared with the same, possibly noncausally related symptoms for all other drugs, based on its pharmacology. The assumption behind this is that associations in the drug_other_—ADR subset will also contain reports where the ADR is not a true ADR but a suspected ADR and could reflect, eg, background noise. Therefore, one may expect a more uniformly distributed TTO compared with a true ADR, and that is what is to be tested. However, when testing against drug—ADR_other_, differences in TTO distributions are inevitable since different ADRs have different pharmacological mechanisms. The low sensitivity found in our previous study was somewhat unexpected and, based on the afore‐mentioned overrepresentation of ADRs with a short TTO, led to the question if the method would perform better when applied to ADRs with a longer TTO. Additionally, our previous study was a proof of concept study investigating only three drugs, resulting in limited generalizability of the results. Therefore, we chose a full database approach in the current study.

The goal of this study was to compare the performance of TTO‐based signal detection of ADRs with a longer TTO with disproportionality analysis in terms of sensitivity and positive predictive value (PPV). We expect that for longer TTOs, the misclassification is less outspoken, and thus, TTO analysis may yield a better performance. To our knowledge, this is the first full database approach investigating the TTO in statistical signal detection using a subset of ADRs with longer TTOs.

## 2 METHODS

In this study, we performed a retrospective analysis of reports of suspected ADRs to detect differences in TTO distributions using a subset of suspected ADRs with a longer TTO and compared the results with disproportionality analysis in terms of sensitivity and PPV. Presence in the Summary of Product Characteristics (SPC) was used as the gold standard to determine if a suspected ADR was a true ADR or not.

### 2.1 Data selection

Data from the spontaneous reporting system maintained by The Netherlands Pharmacovigilance Centre Lareb were used for the study. In the routine assessment of the reports received, presence or absence of the ADR in the SPC is logged by the assessor during assessment of the case report at the drug—ADR level, with the exception of vaccine‐related reports, where presence in the SPC was not logged at all. Therefore, the latter were excluded from this study. Because the objective of the study was to investigate ADRs with a higher latency, only associations with a median TTO of 7 days or more were included. It should be noted that individual reports with a shorter TTO for a certain drug—ADR association were included if the median of the group was 7 days or more. To allow for a proper comparison of both methods, only associations included in the TTO analysis were used in the disproportionality analysis. However, the number of reports included for each association could differ between both methods since reports without a valid TTO were included in the disproportionality analysis but not the TTO analysis. All case reports since the start of reporting to Lareb (1986) until July 2017 were eligible for inclusion. Duplicate reports were excluded, based on the duplicate detection procedure used at Lareb during assessment of individual case reports. Reports from marketing authorization holders (MAH) were excluded. Reports from studies were not explicitly excluded, but because, at the time of data extraction, all reports from studies were received from MAHs only, the exclusion of those reports automatically implied an exclusion of reports from studies. Drugs were classified according to the WHO Anatomical Therapeutic Chemical (ATC) classification system,^13^ using the level of chemical substance (fifth level). ADRs were coded using the preferred terms (PTs) from the Medical Dictionary for Regulatory Activities^14^ (MedDRA, version 19.0).

### 2.2 Disproportionality analysis

The ROR was used as the measure for disproportionality analysis as it is the standard method used at our centre.^15^, ^16^ It is based on a 2 × 2 contingency table as shown in Table 1.

**Table 1:** A 2 × 2 contingency table used for reporting odds ratio (ROR) calculation

	Case Reports with ADR of Interest	Case Reports with All Other ADRs
Case reports with the drug of interest	A*	B*
Case reports with all other drugs	C*	D*

* Each case reports can attribute to only one value in the table. For instance, if the case report has the drug and ADR of interest but also an additional ADR, it is assigned to A, but not to B.

Abbreviation: ADR, adverse drug reaction.

On the basis of Table 1, the ROR and its 95% confidence interval (95%CI) can be calculated using

(1) $$\mathrm{ROR}=\frac{A/B}{C/D}=\frac{\mathrm{AD}}{\mathrm{BC}}$$

(2) $$95\%\mathrm{CI}=e^{\ln\left(\mathrm{ROR}\right)\pm 1.96*\sqrt{\frac{1}{A}+\frac{1}{B}+\frac{1}{C}+\frac{1}{D}}}$$

The ROR was considered statistically significant if the lower limit of the 95%CI was greater than 1.

### 2.3 Time to onset analysis

Differences in the TTO distributions of ADRs were tested using the two‐sample non‐parametric Anderson–Darling (AD) test. This test determines if two samples belong to the same continuous distribution, based on location, dispersion, and skewness.^17^ To investigate the effect of the number of reports per association (N) and TTO values on the performance, several combinations were tested (see Table 2)

**Table 2:** Different combinations of number of reports and median time to onset (TTO) used in the analysis

Number of Case Reports	Median TTO (Days)			
	≥7	≥14	≥30	≥60
≥5	x	x	x	x
≥10	x	x	x	x
≥15	x	x	x	x

For each of the combinations, differences in TTO distributions were tested using two‐sample AD testing (drug—ADR vs drug_other_—ADR). Our previous study was based on a double AD test for each drug—ADR

- drug—ADR versus drug_other_—ADR
- drug—ADR versus drug—ADR_other_

In the interest of between‐study validity, a sensitivity analysis was performed to investigate the effect of the current approach. The sensitivity analysis will be referred to as secondary analysis in order to avoid confusion with sensitivity as the measure of performance. Statistical testing was performed two sided with a significance level of α = .05.

### 2.4 Performance

The performance of both methods was based on the sensitivity and PPV that were defined as described in Equations 3 and 4.

(3) $$\text{Sensitivity}=\frac{\mathrm{TP}}{\mathrm{TP}+\mathrm{FN}}$$

(4) $$\;\mathrm{PPV}=\frac{\mathrm{TP}}{\mathrm{TP}+\mathrm{FP}}$$

Where TP is the number of true positive, FP is the number of false positive and FN is the number of false negative signals (see Table 3 for classification).

**Table 3:** Definitions of true positive, true negative, false positive, and false negative signals

	TTO	ROR
True positive	AD test *P* < .05 and ADR present in SPC*	LL95%CI > 1 and ADR present in SPC
True negative	AD test *P* ≥ .05 and ADR not present in SPC#	LL95%CI ≤ 1 and ADR not present in SPC
False positive	AD test *P* < .05 and ADR not present in SPC^	LL95%CI > 1 and ADR not present in SPC
False negative	AD test *P* ≥ .05 and ADR present in SPC§	LL95%CI ≤ 1 and ADR present in SPC

Abbreviations: AD test, Anderson–Darling test; ADR, adverse drug reaction; LL95%CI, lower limit of the 95%CI of the ROR; ROR, reporting odds ratio; SPC, summary of product characteristics; TTO, time to onset.

For the secondary analysis the definitions are:

* Both AD tests (drug—ADR vs Drug_other_—ADR and vs drug—ADR_other_) *P* < .05 and ADR present in SPC.

# At least one AD test (drug—ADR vs Drug_other_—ADR or vs drug—ADR_other_) *P* ≥ .05 and ADR not present in SPC.

^ Both AD tests (drug—ADR vs Drug_other_—ADR and vs drug—ADR_other_) *P* < .05 and ADR not present in SPC.

§ At least one AD test (drug—ADR vs Drug_other_—ADR or vs drug—ADR_other_) *P* ≥ .05 and ADR present in SPC.

For sensitivity and PPV, interpolated surface plots were generated for both methods to allow for a visual interpretation of the results. Interpolation was based on the Akima algorithm for scattered‐data surface fitting.^18^ Statistical analyses were performed with R statistics version 3.3.2.^19^

## 3 RESULTS

### 3.1 Descriptive statistics

A total of 38 017 case reports, containing 3247 unique drug—ADR associations, were included into the analysis. For the TTO analysis, less reports (n = 29 876) were included due to a lack of information on the TTO, which was missing in 26.5% of the associations. Additional descriptive information is presented in Table 4.

**Table 4:** Descriptive statistics of the case reports used in the study

	Number (n)
Number of case reports	
TTO analysis	29 876
ROR analysis	38 017
Number of associations	
TTO analysis	45 904
ROR analysis	62 440
Number of unique associations	3247
Number of unique drugs*	338
Number of unique suspected ADRs#	484

Abbreviations: ROR, reporting odds ratio; TTO, time to onset.

* Classified according to the Anatomical Therapeutic Chemical (ATC) classification system.

# Coded as MedDRA Preferred Terms (PTs).

### 3.2 Performance

**Figure 1:**
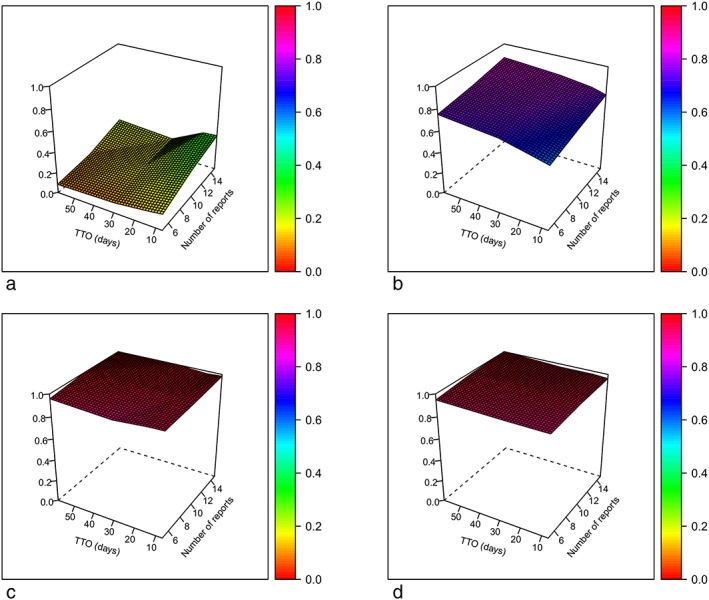
Surface plots of sensitivity and positive predictive value (PPV) after interpolation. (a) Sensitivity for TTO. (b) PPV for TTO. (c) Sensitivity for disproportionality analysis. (d) PPV for disproportionality analysis. PPV = positive predictive value; TTO = time to onset [Colour figure can be viewed at http://wileyonlinelibrary.com]

The sensitivity for the TTO method was low (range 0.08‐0.34) compared with disproportionality analysis (range 0.60‐0.87). In contrast, PPV was similar for both methods (range 0.93‐1.00). Additional information can be found in Table 5. A more detailed analysis showed that both the number of TP and FP signals were three to five times higher for the disproportionality analysis method, whereas the amount of FN signals was approximately two to three times lower. TN signals were similar between groups (data not shown). Interestingly, sensitivity increased with an increasing number of reports for the TTO method, whereas for disproportionality analysis, it increased with both increasing number of reports and increasing TTO (see Figure 1). For the subset with the highest TTO sensitivity (N15_TTO7), 96% of the associations detected by TTO were also detected by disproportionality analysis.

**Table 5:** The number of true positive signals, sensitivity, and positive predictive value (PPV) for each of the combinations of the number of reports and time to onset (TTO)

	True Positive Signals (n)		Sensitivity		PPV	
	TTO	ROR	TTO	ROR	TTO	ROR
N5_TTO7	427	1732	0.16	0.60	0,96	0,93
N10_TTO7	283	783	0.27	0.68	0,97	0,96
N15_TTO7	193	471	0.34	0.74	0,98	0,96
N5_TTO14	269	1330	0.14	0.64	0,95	0,93
N10_TTO14	175	580	0.24	0.71	0,97	0,96
N15_TTO14	126	351	0.33	0.78	0,98	0,97
N5_TTO30	100	780	0.10	0.70	0,93	0,94
N10_TTO30	54	309	0.17	0.77	0,98	0,97
N15_TTO30	26	169	0.18	0.82	1,00	0,97
N5_TTO60	47	507	0.08	0.75	0,96	0,95
N10_TTO60	21	191	0.12	0.80	1,00	0,98
N15_TTO60	14	99	0.23	0.87	1,00	1,00

Abbreviation: ROR, reporting odds ratio.

### 3.3 Secondary analysis

**Figure 2:**
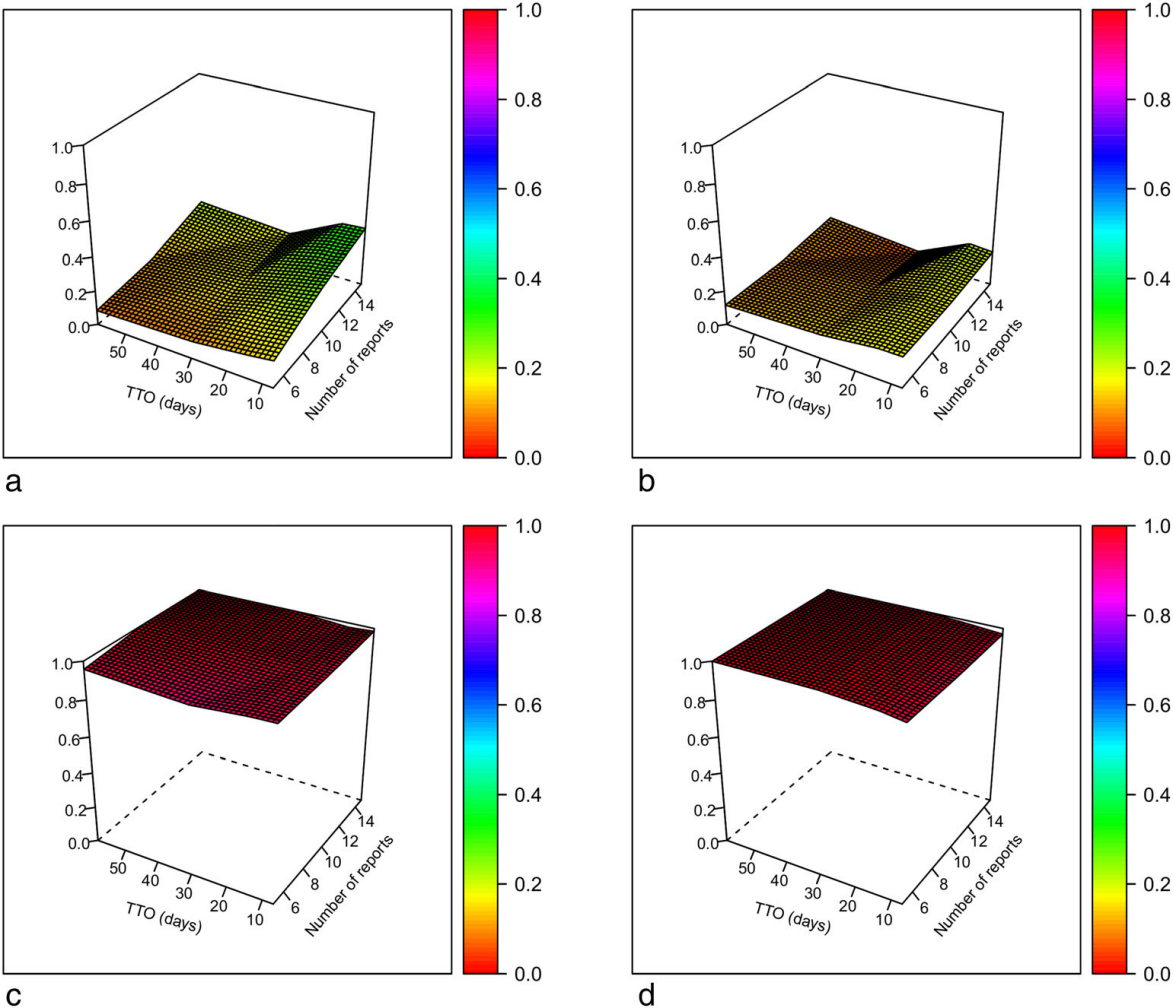
Surface plots of sensitivity and positive predictive value (PPV) for the secondary analysis. (a) Sensitivity for original TTO analysis. (b) Sensitivity for secondary analysis. (c) PPV for original TTO analysis. (d) PPV for secondary analysis. PPV = positive predictive value; TTO = time to onset [Colour figure can be viewed at http://wileyonlinelibrary.com]

The secondary analysis showed a similar pattern for sensitivity and PPV as the original analysis (see Figure 2). However, absolute values for sensitivity were in general slightly lower for the secondary analysis (0.07‐0.21).

## 4 DISCUSSION

In this follow‐up study, we investigated the performance of a previously developed TTO signal detection method using the two‐sample AD test applied to ADRs with a longer TTO and compared it with disproportionality analysis. The major reason for conducting this study was the previous finding that the majority of TTOs was 1 to 2 days, possibly limiting discriminative power of the statistical test.

Reports from MAHs were excluded because it is our experience they are more often poorly documented than reports received directly by Lareb. In addition, presence of the ADR in the SPC is not logged since reports from MAHs are not assessed manually. There was a substantial amount of case reports with missing TTO values. However, we do not have any indication that this occurs on a selective basis (eg, for certain types of drugs and/or ADRs).

The results show that sensitivity was lower for the TTO method, whereas PPV was similar and although there was a small number of associations that was detected by the TTO only, this did not apply to particular clinical entities. The similarity in PPV between the TTO method and disproportionality analysis can be explained by the three to five times higher FP value for the ROR method (thereby nullifying its three to five times TP advantage over the TTO method). The secondary analysis showed similar results although in general, sensitivity was slightly lower than in the original analysis. In signal detection using spontaneously reported data, it is most important not to miss a true signal as it is a timely detection of a signal. Therefore, we compared performance in terms of sensitivity and PPV, deliberately neglecting possible differences in specificity. The observation that sensitivity for the TTO method increases with increasing number of reports per association (n) cannot be explained unambiguously but may be a statistical artefact since more reports lead to a larger test sample and subsequently to more statistical power.

Previous similar studies by others were performed using the two‐sample Kolmogorov–Smirnov test.^3^, ^4^, ^6^ However, the AD test has generally more power and is more sensitive to differences in shift, scale or symmetry. In addition, it is better at detecting small differences, even when samples sizes are larger.^17^, ^20^ Given these facts, we considered the AD test to be more appropriate for our study.

The major strength of this study is its full database approach covering a wide range of drug—ADR associations. Compared with methods where a subset of drugs is used in the analysis, this approach reduces selectivity in the results and increases generalizability. On the other hand, databases containing spontaneous reports show substantial differences, and a similar approach for a different database may result in a better performance.^21^

Additionally, the comparison between the statistically stricter approach and the more tolerant approach (both tests significant versus only one test significant for a TP signal respectively) and the fact that the results were similar, increases the between‐study validity.

As mentioned in our previous study, the use of the SPC as the gold standard (particularly in finding new, previously undocumented ADRs) has its drawbacks and could influence the results of this study by introducing misclassification because presence in the SPC does not necessarily imply a causal relationship between drug and ADR. The opposite is also true since absence in the SPC does not necessarily imply absence of causality. Moreover, the SPC lists both ADRs identified prior to and after market authorization, where the former may be preferentially reported immediately after authorization, artificially influencing some counts. Nevertheless, we consider the use of the SPC valid because (a) it is the only variable regarding causality that is consistently present for each drug—ADR association in our database since it is logged during manual assessment of the report; (b) if any misclassification occurs, it is most likely to be nondifferential and would therefore not account for any major differences between the two methods investigated. A second limitation of this study is the exclusion of vaccine related reports due to the lack of information about presence of the ADR in the SPC in our database. Since previous studies related to vaccine report by others^3^, ^4^, ^5^, ^6^ showed promising results, it would be very valuable to investigate these reports in our database. Recently, Lareb has taken a new database into production providing the possibility to add information about presence in the SPC to vaccine related reports. In due time, this information can be used to perform similar studies for these type of reports. Finally, some selection bias may have been introduced. Since a positive ROR (or any other measure of disproportionality) may have been the trigger for a signal in the past, we cannot rule out that this trigger resulted in the inclusion of the ADR into the SPC. So theoretically, the ROR could have contributed directly to the outcome (SPC) introducing selection bias. We think however, that this will account for a limited number of drug—ADR associations and will not have had a serious influence on the results of our study, if any at all.

The results of the secondary analysis were in line with those of the original analysis, with similar patterns in sensitivity and PPV emerging from the surface plots. The lower absolute value for sensitivity compared with the original analysis can be explained by the fact that both AD tests had to be statistically significant to allow for a true positive signal in the secondary analysis, resulting in a decreased number of true positive signals. Additionally, the fact that only one of both tests had to be statistically significant to allow for a false negative signal resulted in an increase in false negative signals and therefore a corresponding decrease in sensitivity.

One final issue to consider is the fact that databases containing spontaneous reports are cross‐sectional. It is well‐known that this type of data can be subject to various sorts of bias including recall bias. This type of bias may result in a less precise estimation of the TTO by the reporter and subsequently to a certain degree of randomness in the collected data, thereby decreasing statistical power. Additionally, TTO clustering may occur depending on the unit reported. For instance, both a TTO of 6 and 8 days may be reported as 1 week. On the basis of the results of the current study, the TTO method does not perform well enough to replace disproportionality analysis as a screening tool. However, the results of the AD test may be useful as a parameter in a prediction model‐based screening approach similar to the one we published recently.^22^

## 5 CONCLUSIONS

The results of our study show that TTO‐based signal detection, restricted to a dataset containing ADRs with a longer TTO only, cannot replace disproportionality analysis as a screening method. This may in part be due to the presence of several types of bias known to occur in spontaneous reporting.

## ETHICS STATEMENT

The authors state that no ethical approval was needed.

## CONFLICT OF INTEREST

The authors declare no conflict of interest.
